# Thermo-Hydraulic Analysis of Heat Storage Filled with the Ceramic Bricks Dedicated to the Solar Air Heating System

**DOI:** 10.3390/ma10080940

**Published:** 2017-08-12

**Authors:** Magdalena Nemś, Artur Nemś, Jacek Kasperski, Michał Pomorski

**Affiliations:** Faculty of Mechanical and Power Engineering, Wroclaw University of Science and Technology, Wybrzeze Wyspianskiego 27, 50-370 Wroclaw, Poland; artur.nems@pwr.edu.pl (A.N.); jacek.kasperski@pwr.edu.pl (J.K.); michal.pomorski@pwr.edu.pl (M.P.)

**Keywords:** heat storage, ceramic brick material, solar air space heating system

## Abstract

This article presents the results of a study into a packed bed filled with ceramic bricks. The designed storage installation is supposed to become part of a heating system installed in a single-family house and eventually to be integrated with a concentrated solar collector adapted to climate conditions in Poland. The system’s working medium is air. The investigated temperature ranges and air volume flow rates in the ceramic bed were dictated by the planned integration with a solar air heater. Designing a packed bed of sufficient parameters first required a mathematical model to be constructed and heat exchange to be analyzed, since heat accumulation is a complex process influenced by a number of material properties. The cases discussed in the literature are based on differing assumptions and different formulas are used in calculations. This article offers a comparison of various mathematical models and of system operating parameters obtained from these models. The primary focus is on the Nusselt number. Furthermore, in the article, the thermo-hydraulic efficiency of the investigated packed bed is presented. This part is based on a relationship used in solar air collectors with internal storage.

## 1. Introduction

Energy storage is becoming an increasingly important issue. The growing world economy causes an increased demand for energy in its various forms. Such situation has been long predicted, inter alia by Starr, who suggested, in the 1990s, a graph representing an increasing role that energy storage is going to play in the global energy balance between 1980 and 2016 [1]. According to the graph, stored energy should currently cover approximately 20% of global demand and its quantity should be comparable with the quantity of energy produced from conventional sources, such as coal. The same graph forecasts that, around 2040, stored energy should account for approximately 36% global demand.

Thermal energy storage (TES) systems are mainly coupled with sources of heat whose availability and intensity vary in time, i.e., mostly with renewable energy sources, and especially with solar energy systems. Heat storage is also used in industrial waste heat recovery systems. Although charging and discharging processes usually take place at the same location, mobile heat storage units are also sometimes used to deliver heat to demand sites located at distances of up to several kilometers [2].

Current research trends focus on phase change materials (PCM) as the most promising technology for heat energy accumulation processes [3]. Some works, as well as this article, are devoted to the issue of energy storage in sensible heat storage materials. Stone thermal storage units offer many advantages, including simple design and easy maintenance, low price, and safe operation. They can also work in a broad temperature range. Research into stone heat storage enjoyed much popularity in the 1980s and 1990s, and resulted in many publications written at that time. This research explored inter alia the issue of pressure drop in packed beds with large-scale filling elements [4,5], experiments with a high-temperature accumulator (up to 700 °C) [6], and a solar thermal collector with internal rock bed storage [7]. Current research dedicated to sensible heat storage materials focuses on finding operating characteristics for packed beds having a particular design and on their specific industrial applications, e.g., as a storage system in a solar power plant [8,9] or in a greenhouse [10]. Solutions dedicated to building heating systems, e.g., to achieve level temperatures on a daily basis, are less frequently proposed [11].

**Figure 1:**
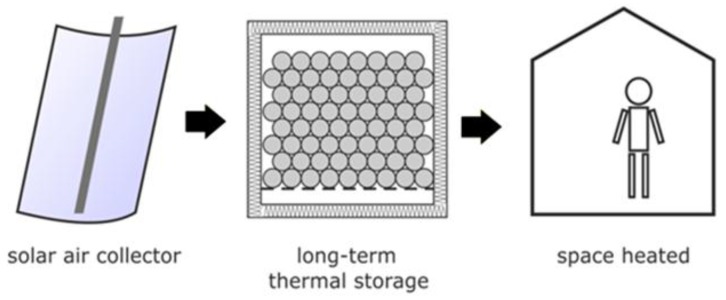
The concept of a solar heating system.

Further part of the article offers the results of experiments on a packed bed filled with ceramic bricks. In the future, the bed is supposed to be charged with the air coming from a concentrated solar thermal collector system adapted to climate conditions in Poland (Figure 1). In the concept design of the heating system, the packed bed functions as a long-term heat storage unit located inside the building. The unit’s planned volume is dedicated to a house constructed using energy-saving technology and is initially estimated at 8 m^3^. 

The choice of ceramic bricks as the filling material was dictated by several reasons. Structural stability can be provided more easily to a large bed filled with bricks than to a bed filled with, e.g., crushed stone or pebbles. Brick is also an easily available material and has good thermal properties. Additionally, brick is resistant to high temperatures and tolerates a high number of charge/discharge cycles. Brick does not emit any harmful gases in high temperatures, which is important, as the storage unit is to be located inside a residential house. 

## 2. Thermo-Hydraulic Efficiency

The analysis of a rock heat storage system normally covers finding the amount of energy accumulated and the efficiency of the accumulation process. The authors of this article suggest that the charging process efficiency be calculated analogically to “*η_eff_*− effective efficiency” (Equation (1)) proposed in 1990 by Cortes and Piacentini [12]:

(1) $$\eta_{eff}=\frac{Q_{u}-\frac{p_{m}}{g}}{I\cdot A}$$

where *Q_u_* is the useful heat gain, *p_m_* is the mechanical energy consumed for propelling air through collector, *g* is the constant, *I* is the intensity of solar radiation and *A* is the area of absorber.

Mechanical energy consumption (*p_m_*) for propelling air through the duct (Equation (2)) refers to mechanical energy loss due to flow resistance:

(2) $$p_{m}=\frac{\dot{m}\cdot ∆p}{\rho }=\dot{V}\cdot ∆p$$

where $\dot{m}$ is the mass flow rate, *Δp* is the pressure drop across collector length and *ρ* is the density of fluid. Conversion efficiency *g* is calculated from the following relationship:

(3) $$g=\eta_{F}\eta_{E}\left(1-\zeta_{t}\right)\chi_{c},$$

where *η_F_* is the efficiency of the fan, *η_E_* is the electric engine, *ζ_t_* is the electric transmission loss coefficient and $\chi_{c}$ is the efficiency of thermal-electric conversion process. The assumed value was *g* = 0.18 in Argentina [12] and *g* = 0.20 in India [13]. This efficiency, nowadays more commonly referred to as the thermo-hydraulic efficiency, is frequently used in research papers as a means to analyze the work of solar air collectors with internal storage [14] or with additional elements improving heat exchange [15,16,17]. The relationship includes the amount of energy related to pressure drop in the system due to the flow resistance of the working medium. 

The authors of this paper suggest that the thermo-hydraulic efficiency of the process of heat accumulation in a packed bed (*η_t−h_*) be described with Equation (4), and be defined as: the ratio between the amount of energy absorbed by the packed bed ${\dot{Q}}_{s}$ minus the energy lost due to the flow resistance of the working medium in the packed bed and the total amount of energy ${\dot{Q}}_{c}$ which could be potentially absorbed by the packed bed:

(4) $$\eta_{t-h}=\frac{\dot{Q_{s}}-\frac{\dot{V}\cdot ∆p_{c}}{\eta_{fan}}}{{\dot{Q}}_{c}},$$

where $\dot{Q_{s}}$ is the heat flux absorbed by the filler material of the packed bed, $\dot{V}$ is the volumetric flow rate, *Δp_c_* is the total pressure drop in the system, *η_fan_* is the efficiency of the fan and $\dot{Q_{c}}$ is the heat input flux. Conversion efficiency (Equation (3)) was replaced with the total efficiency of the fan. Its assumed value is 0.7.

## 3. Mathematical Model

**Figure 2:**
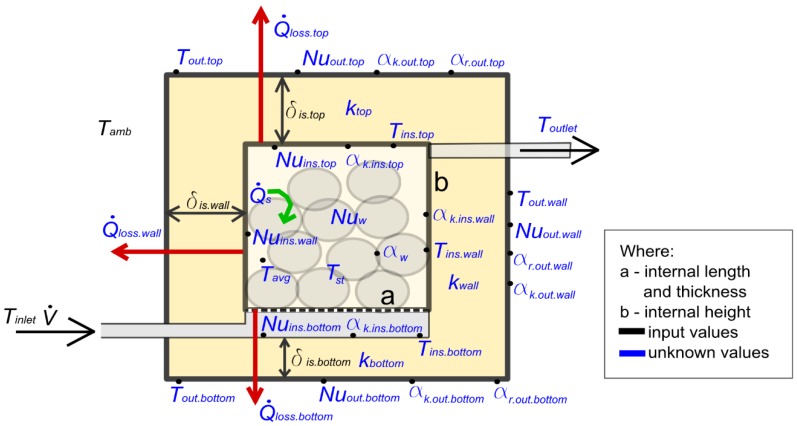
Scheme of the heat storage device with indicated input and calculation parameters.

Calculating thermo-hydraulic efficiency for the process of charging the packed bed required the construction of a mathematical model. The model involved balance calculations of heat input and heat output to/from the heat storage device with consideration to its geometry, as described in Figure 2. Each time step was assumed to be characterized by some steady conditions. The calculations were performed with the use of a steady heat exchange equation for each step. Table 1 shows the steady parameters assumed in the calculations.

Heat flux absorbed by the filler material of the packed bed (${\dot{Q}}_{s}$) is equal to heat input flux (${\dot{Q}}_{c}$) minus heat loss through the walls of the packed bed (${\dot{Q}}_{loss}$). The accumulation process does not include heat output flux (${\dot{Q}}_{L}$), which therefore has a value of zero in general Equation (5):

(5) $${\dot{Q}}_{s}={\dot{Q}}_{c}-{\dot{Q}}_{L}-{\dot{Q}}_{loss}.$$

Equation (6) describes the heat input flux:

(6) $${\dot{Q}}_{c}=\dot{m}c_{p_{air}}\left(T_{inlet}-T_{outlet}\right),$$

where $\dot{m}$ is the mass flux of the air passing through the packed bed, *c_p.air_* is the heat proper of air at specific pressure, *T_inlet_* is the air inlet temperature, and *T_outlet_* is the air outlet temperature.

The flux of heat accumulated in the packed bed is described with Equation (7):

(7) $${\dot{Q}}_{s}=\frac{m_{st}\cdot c_{pst}\cdot ∆T_{st}}{\tau },$$

where *m_st_* is the heat storage mass, *c_pst_* is the specific heat of the filler material, and *ΔT_st_* is the temperature rise in the packed bed. The accumulated heat flux is equal to the heat flux transferred to the filler material (Equation (8)):

(8) $${\dot{Q}}_{s}=\alpha_{w}\cdot S\cdot \left(T_{avg}-T_{st}\right),$$

where *α_w_* is the heat-transfer coefficient, *S* is the area of the filler material, and *T_avg_* is the average temperature, described with Equation (9):

(9) $$T_{avg}=\frac{T_{inlet}+T_{outlet}}{2}.$$

The heat transfer coefficient is described with Equation (10):

(10) $$\alpha =\frac{\lambda \cdot Nu}{x}$$

where *λ* is the thermal conductivity coefficient, *Nu* is the Nusselt number and *x* is the specific dimension. When calculating the coefficient of heat-transfer into the storage material *α_w_*, specific parameter *x* was assumed to be *D_mat_*, which is the diameter of a sphere having the same volume *V_mat_* as the volume of a single brick, calculated as in Equation (11).

(11) $$D_{mat}=\sqrt[{3}]{\frac{6\cdot V_{mat}}{\pi }}$$

Equation (10) served to establish the coefficient of heat transfer from the air passing inside the packed bed to the surface of the bed walls. Specific parameter *x* used to establish *α_k.ins.wall_* was the height of the packed bed *b*, while the characteristic parameter used to establish *α_k.ins.top_* and *α_k.ins.bottom_* was the width of the packed bed *a*.

Equation (10) was also used to find the convective heat-transfer coefficient on the inside of the storage device *α_k.out_*. The *α_k.out.wall_* was calculated from *x = b + 2·δ_is.top−bottom_*, while the *α_k.out.top_* and *α_k.out.bottom_* were calculated from *x = a + 2·δ_is.wall_*.

Nusselt number is the function of Reynolds number (*Re*) and Prandtl number (*Pr*):

(12) $$Nu_{w}=A\cdot Re^{B}\cdot Pr^{C}.$$

Constants *A*, *B*, and *C* in this formula describe the process of heat transfer into the filler material of the storage device.

Formula (13) describes the heat loss flux from the packed bed:

(13) $${\dot{Q}}_{loss}=\left(k\cdot A\right)\cdot \left(T_{avg}-T_{amb}\right),$$

where *k* is the overall heat transfer coefficient, *A* is the area, and *T_amb_* is the ambient temperature.

After including the area of heat loss from the packed bed, the formula becomes Equation (14):

(14) $$\begin{array}{c} {\dot{Q}}_{loss}={\dot{Q}}_{loss.top}+{\dot{Q}}_{loss.bottom}+{\dot{Q}}_{loss.wall}=[k_{top}{\left(a+2\delta_{is_{wall}}\right)}^{2}+k_{bottom}{\left(a+2\delta_{is_{wall}}\right)}^{2}]\\ +k_{wall}\cdot [4\cdot \left(a+2\delta_{is_{wall}}\right)\cdot b+\left(2\delta_{is_{top-bottom}}\right)]\left(T_{avg}-T_{amb}\right). \end{array}$$

Equations (7), (8) and (14) served to find heat transfer flux $\dot{Q}$*_s_* and temperatures: *T_outlet_* and *T_st_*.

After Equations (6) and (14) were substituted into Equation (5), the result was:

(15) $$\begin{array}{c} {\dot{Q}}_{s}=\dot{m}c_{p}\left(T_{inlet}-T_{outlet}\right)-[k_{wall}\cdot \left[4\cdot \left(a+2\delta_{is_{wall}}\right)\cdot \left(b+2\delta_{is_{top-bottom}}\right)\right]\\ +k_{bottom}(a+{2\delta_{is_{wall}})}^{2}+k_{top}{\left(a+2\delta_{is_{wall}}\right)}^{2}]\left(T_{avg}-T_{amb}\right). \end{array}$$

The overall coefficients of heat transfer through the housing of the packed bed are calculated from Equation (16) [18]:

(16) $$k=\frac{1}{\frac{1}{\alpha_{k}}+\frac{\delta_{wall1}}{\lambda_{wall1}}+\frac{\delta_{is}}{\lambda_{is}}+\frac{\delta_{wall2}}{\lambda_{wall2}}+\frac{1}{\alpha_{k}+\alpha_{r}}},$$

where *α_k_* is the convective heat-transfer coefficient, and *α_r_* is the radiant heat-transfer coefficient.

To find the coefficient of heat transfer through the side wall *k_wall_*, three coefficients were used: the coefficient of heat transfer on the inside wall *α_k.ins.wall_*, as well as the convective coefficient *α_k.out.wall_* and the radiant coefficient on the outside wall. Due to its negligible influence on the obtained heat flux, the resistance caused by the steel sheet used in the housing of the packed bed was disregarded $\frac{\delta_{wall1}}{\lambda_{wall1}}$ and $\frac{\delta_{wall2}}{\lambda_{wall2}}$—value *δ_is_* was substituted with *δ_is.wall_* and *λ_is_* of the insulating material. The same procedure was performed for the top surface, whose coefficient *k_top_* was calculated by substituting *α_k.ins.top_*, *α_k.out.top_*, *α_r.out.top_* and by assuming insulation thickness *δ_is.top-bottom_* and for the bottom surface, in which *k_bottom_* depended on *α_k.ins.bottom_*, *α_k.out.bottom_* , *α_r.out.bottom_* and on insulation thickness *δ_is.top−bottom_*.

Convective heat-transfer coefficient is calculated as in Equation (10), while radiant heat-transfer coefficient is calculated from Equation (17):

(17) $$\alpha_{r}=\frac{\varepsilon_{wall}\cdot \sigma \cdot \left(T_{out}^{4}-T_{amb}^{4}\right)}{T_{out}-T_{amb}},$$

where *ɛ_wall_* is the emissivity of the plate, *σ* is the Stefan–Boltzmann constant and *T_out_* is the temperature of the packed bed’s outside surface. The radiant coefficient of heat absorption from the side wall *α_r.out.wall_* was calculated by assuming surface temperature *T_out.wall_*. Coefficients *α_r.out.top_* and *α_r.out.bottom_* were calculated by assuming temperatures *T_out.top_* and *T_out.bottom_*, respectively.

An assumption was made that on the outside wall of the storage device’s housing, heat is transferred by radiance described with Formula (17) and by natural convection. Therefore, the convective heat-transfer coefficient on the outside of the packed bed was calculated from the relationship between Nusselt number and Prandtl and Grashoff numbers (Equation (18)):

(18) $$Nu_{out}=C\cdot {\left(Gr\cdot Pr\right)}^{n},$$

where according to Kostowski [19]:

Gr·Pr < 10^−3^, *C* = 0.5, *n* = 0
10^−3^ < Gr·Pr<500, *C* = 1.18, *n* = 1/8
$500\leq Gr\cdot Pr<2\cdot 10^{7}$, *C* = 0.54, *n* = 1/4
$2\cdot 10^{7}\leq Gr\cdot Pr$, *C* = 0.135, *n* = 1/3.

For such case, the Grashoff number was calculated from Equation (19):

(19) $$Gr=\frac{g\cdot x^{3}\frac{1}{\frac{T_{amb}+T_{out}}{2}}\left(T_{out}-T_{amb}\right)}{\nu^{2}},$$

where *g* is the gravitational acceleration, *x* is the specific dimension, and *ν* is the coefficient of kinematic viscosity.

The Nusselt number was established for the outside surface of the side wall *Nu_out.wall_* by assuming specific dimension *x* to be *b + 2·δ_is.top-bottom_* and the temperature of the outside surface to be *T_out.wall_*. At the same time, *Nu_out.top_* was calculated by assuming *x* to be *a + 2·δ_is.wall_* and the temperature of the outside surface to be *T_out.top_*, and the assumptions for the bottom surface were analogically *x = a + 2·δ_is.wall_* and temperature *T_out.bottom_*.

The convective heat-transfer coefficient on the inside of the storage device was calculated from the *Nu_ins_* Equation (20) [19] describing heat transfer during laminar flow around the plate:

(20) $$Nu_{ins}=0.593\cdot Re^{0.5},$$

where, for the sidewall surface, the value of *Nu_ins.wall_* was established by substituting specific parameter *x* with internal height of the bed *b*, and to calculate the Nusselt number for the top surface *Nu_ins.top_* and for the bottom surface *Nu_ins.bottom_*, *x* was substituted with base length *a*.

The model included calculations made to ensure that the assumed temperatures on the walls of the housing are correct. The flux of heat loss through each of the housing layers was assumed to be constant:

(21) $${\dot{Q}}_{loss}=\left(T_{avg}-T_{ins}\right)\cdot \alpha_{k_{ins}}\cdot A_{ins},$$

(22) $${\dot{Q}}_{loss}=\left(T_{out}-T_{amb}\right)\cdot \left(\alpha_{k.out}+\alpha_{r.out}\right)\cdot \frac{A_{ins}+A_{out}}{2},$$

(23) $${\dot{Q}}_{loss}=\left(T_{ins}-T_{out}\right)\cdot \left(\frac{\delta_{wall1}}{\lambda_{wall1}}+\frac{\delta_{is}}{\lambda_{is}}+\frac{\delta_{wall2}}{\lambda_{wall2}}\right)\cdot A_{out},\;$$

where *T_ins_* is the temperature of the wall on the inside, *A_ins_* is the area of the inside wall and *A_out_* is the temperature of the wall on the outside. *A_ins_* for the sidewall surface was described as *A_ins.wall_* = 4∙*a*∙*b*, and *A_out_* as *A_out.wall_* = 4∙(*a* + 2∙*δ_is.wall_*)∙(*b* + 2∙*δ_is.top.bottom_*). Meanwhile, *A_ins_* for the top and bottom surface was describe as *A_in.top.bottom_* = *a*^2^, and *A_out_* as *A_out.top.bottom_* = (*a* + 2∙*δ_ins.wall_*)^2^.

Equations (21)–(23) were used to find heat loss fluxes from sidewall, top and bottom surfaces of the packed bed. Values *T_ins_*, *T_out_*, *α_k.ins_*, *α_k.out_*, *α_r.out_* and *δ_is_* for each surface were assumed in accordance with the labels in Figure 2, forming a set of nine equations (three per a loss flux in each direction: $\dot{Q}$*_loss.wall_*, $\dot{Q}$*_loss.botttom_*, and $\dot{Q}$*_loss.top_*), which allow the calculation of unknown loss fluxes, $\dot{Q}$*_loss.wall_*, $\dot{Q}$*_loss.botttom_*, and $\dot{Q}$*_loss.top_*, and of the temperatures on the walls of the bed, *T_ins.wall_*, *T_out.wall_*, *T_ins.bottom_*, *T_out.bottom_*, *T_ins.top_*, and *T_out.top_*.

The working medium was air considered as a semi ideal gas. A set was obtained of 33 balance equations and 33 unknown values. The unknown values are:

- temperatures: *T_outlet_*, *T_st_*, *T_avg_*, *T_ins.wall_*, *T_out.wall_*, *T_ins.bottom_*, *T_out.bottom_*, *T_ins.top_*, and *T_out.top_*;
- heat fluxes: $\dot{Q}$*_s_*, $\dot{Q}$*_loss.wall_*, $\dot{Q}$*_loss.botttom_*, and $\dot{Q}$*_loss.top_*;
- the Nu numbers: *Nu_w_*, *Nu_out.wall_*, *Nu_ins.wall_*, *Nu_ins.bottom_*, *Nu_out.bottom_*, *Nu_ins.top_*, and *Nu_out.top_*;
- heat-transfer coefficients: *α_w_*, *α_k.out.wall_*, *α_k.ins.wall_*, *α_r.out.wall_*, *α_k.ins.bottom_*, *α_k.out.bottom_*, *α_r.out.bottom_*, *α_k.ins.top_*, *α_k.out.top_*, and *α_r.out.top_*; and
- overall heat transfer coefficients: *k_wall_*, *k_bottom_*, and *k_top_*.

To provide an iterative solution to this system of equations, commercially available Mathcad 15.0 software was used. 

The effectiveness of the accumulation process should include pressure drop in the system, which is related to the flow resistance due to the shape and the filling factor of the packed bed, analogically to the calculations for a solar thermal collector [20,21]. To find total pressure drop *Δp_c_* using the packed bed’s measurement system, it was necessary to calculate the pressure drop in the bed itself and in the outlet channel, as well as the local pressure drop due to the system’s geometry. Friction loss factor in the packed bed *f_m_* [14] is described by Equation (24):

(24) $$f_{m}=150\cdot \left[\frac{1-\varepsilon_{st}}{Re_{st}}\right]+1.75$$

where *ɛ_st_* is the filling factor of the packed bed with air. Its solution required calculating the Reynolds number for the flow of the working medium through the packed bed, from Formula (25):

(25) $$Re_{st}=\frac{D_{e}\cdot \rho_{air}\cdot w}{\mu },$$

where *D_e_* is the specific dimension of the filler material, *w* is the flow velocity of the working medium and *µ* is the coefficient of dynamic viscosity. The filler material’s specific dimension *D_e_* [14] is:

(26) $$D_{e}=\frac{2}{3}\cdot \frac{\varepsilon_{st}\cdot D_{mat}}{1-\varepsilon_{st}},$$

The *D_e_* dimension used in the pressure drop formulas included equivalent diameter of the filler material *D_mat_*. According to the literature, the equivalent diameter of the filler material can be approximated by the diameter of a sphere. Therefore, its calculation required finding the mass of bricks and counting the number of filler elements. The filling factor of the packed bed with air, which is required to solve Equation (22), can be calculated from Equation (27) [14]:

(27) $$\varepsilon_{st}=\frac{V_{c}-nV_{mat}}{V_{c}},$$

where *V_c_* is the total bed volume. 

Pressure drop in the bed, which results from transforming the formulas provided in [14,22], is described by:

(28) $$∆p_{st}=\frac{2\cdot \rho_{air}\cdot f_{m}\cdot {\dot{V}}^{2}\cdot b\cdot \left(\dot{1}-\varepsilon_{st}\right)}{B^{2}\cdot D_{e}\cdot \varepsilon_{st}^{3}},$$

where *B* is the bed’s section area and *b* is the bed’s height. Calculating pressure drop in the outlet channel firstly required finding the Reynolds number, according to Equation (29):

(29) $$Re_{k}=\frac{\rho_{air}\cdot v_{k}\cdot D_{k}}{\mu },$$

where *v_k_* is the velocity in the channel, and *D_k_* is the diameter of the channel. The next step consisted in using the friction coefficient ξ formula, which was assumed for the turbulent flow, according to Blasius formula [23]:

(30) $$\xi =\frac{0.316}{Re_{k}^{0.25}}.$$

Pressure loss due to friction during turbulent flow through a straight duct of any and uniform section, as described with the Darcy–Weisbach relationship [24], is defined as:

(31) $$\Delta p_{k}=\xi \cdot \frac{L_{k}\cdot v_{k}^{2}\cdot \rho_{air}}{4\cdot R_{h}\cdot 2},$$

where *L_k_* is the length of the duct, and *R_h_* is the hydraulic diameter.

In order to calculate local pressure drop due to the change of the shape and direction of flow, first it is necessary to assume a local resistance coefficient. According to tables provided in [25], a coefficient was selected for an elbow pipe with additional change of the section area of the elements: ζ = 1.24. Local pressure loss should be in this case proportional to the dynamic pressure of the fluid stream (31):

(32) $$\Delta p_{m}=\zeta \cdot \frac{v_{\text{ś}r}^{2}\cdot \rho_{p}}{2}.$$

Thus, the total pressure drop in the system is described by Equation (33):

(33) $$∆p_{c}=∆p_{st}+∆p_{k}+∆p_{m}.$$

### The Nusselt Number

The literature offers many formulas for calculating the Nusselt number for the flow around the storage material. Out of the available mathematical formulas, the authors chose to use those which had been applied for the filler material in the form of spheres, or which had an equivalent diameter calculated. In each of the cases, the working medium was air, considered as a semi ideal gas, for which the assumed Prandtl number was 0.7. Table 2 shows eight formulas along with their conditions of use (if such had been provided).

With the same boundary conditions selected, the above formulas provide different results. By comparing the results of actual experiments with the results from mathematical models, it will be possible to indicate the formula which best characterizes the process of thermal energy storage in ceramic bricks.

## 4. Experimental Set-Up

**Figure 3:**
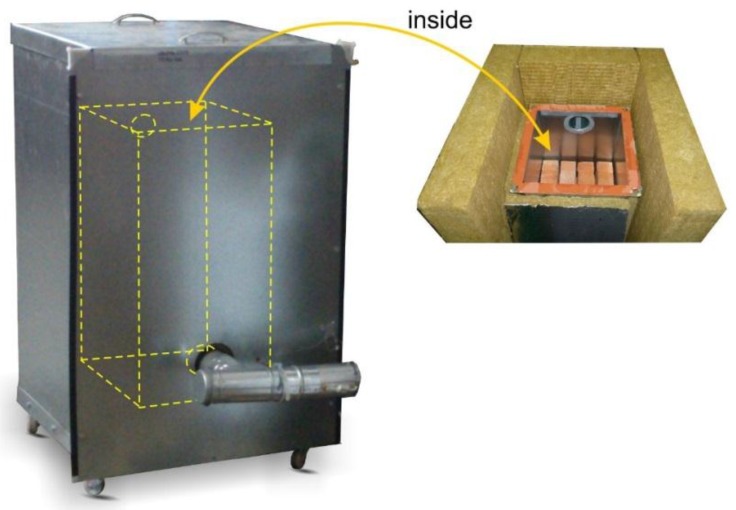
Experimental set-up for examining heat storage process in the rock bed.

Figure 3 shows the experimental set-up used in the experiments on the process of heat storage. The experimental set-up comprises two metal, cuboid-shaped housings, with mineral wool inserted between them. The chosen filler material is placed in the inner housing. Both housings are made of zinc coated sheets between 1 mm and 3 mm in thickness. The remaining dimensions and operating parameters of the packed bed are provided in Table 1. 

### 4.1. The Operating Principle and the Measuring Apparatus

**Figure 4:**
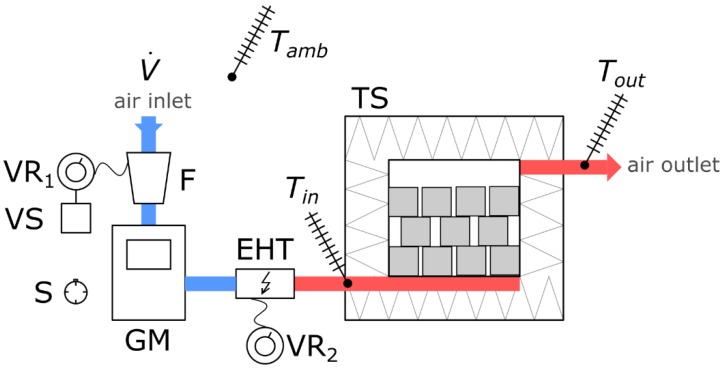
Schematic diagram of the experimental set-up for examining heat storage process.

Figure 4 shows a schematic diagram of the experimental set-up with the measuring apparatus.

Air stream $\dot{V}$ is forced into the system with fan F. The fan is powered by autotransformer VR1, which allows changing the stream of the flowing air. Autotransformer VR1 is additionally connected to voltage regulator VS. The air then flows through gas flowmeter GM, which allows reading the volume of the flowing medium over time measured by stop watch S. The air forced by fan F flows through electric heater EHT having a maximum power of 1.7 kW, powered from autotransformer VR2. This allows regulating inlet temperature *T_in_*. The settings of VR2 are determined experimentally and depend on the stream of airflow and on ambient temperature *T_amb_*. In order to flatten the velocity field, warmed airflows into packed bed TS through symmetrically distributed intake apertures, where it gives off heat to the filler material. Cooled air having temperature *T_out_* flows through an outlet channel located in the upper part of packed bed TS and is dissipated in the environment. The system is open-cycle. 

Temperature measurement is performed with K-type thermocouples. The measured temperatures include: ambient temperature *T_amb_*, inlet temperature *T_in_* and outlet temperature *T_out_*. The results were recorded at 60-s intervals on Lumel KD7 automatic data logger. 

### 4.2. Experimental Tests and Uncertainty Analysis

The tests of heat storage process in ceramic brick were performed for two airflow rates and two different inlet temperatures. Both the airflow stream and the values of temperatures at the inlet of the medium to the packed bed were selected from a perspective of integrating the packed bed with a concentrated solar collector as part of a heating system installed in a single-family house. The parameters were selected previously, according to the analyses of the solar heater, as offered in [20,21].

Initial parameters for the first and for the second experiment are shown in Table 2. Ambient temperature for both measurements was 18 °C. In the second experiment, for a smaller airflow, the charging process was performed on the packed bed which was not fully discharged. The temperature of the packed bed was 28 °C, which corresponds to conditions frequently encountered in the target system. During the experiments, the end of the heat storage process was marked by the absolute increment of outlet temperature over the 10-minute period being ΔT < 2 °C for the first airflow, and ΔT < 1 °C for the second airflow. 

**Figure 5:**
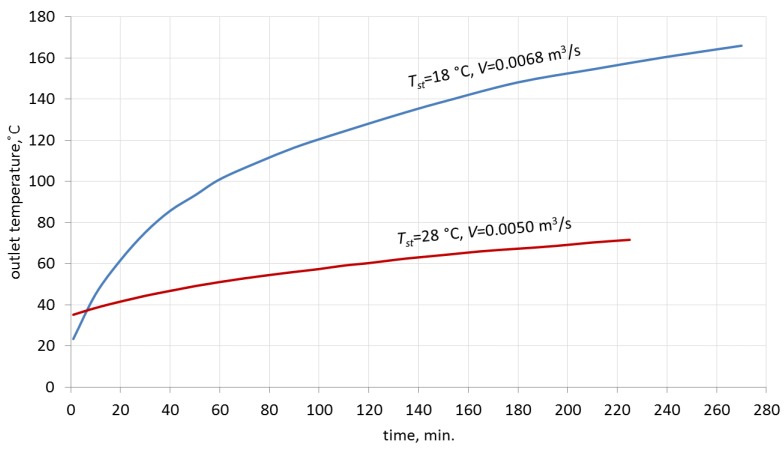
Change of outlet temperature as a function of charging time for two examined airflow rates.

Figure 5 illustrates the changes in the outlet temperature of air over time. The significantly different values of temperature at the inlet to the packed bed result in considerably different dynamics of the process of heat absorption by the packed bed in the first hour of the process. This fact is of importance for the construction of a model which will accurately represent the character of the process. The shape of the characteristic curve in the first hour of the charging process is of great importance also because of the planned integration of the packed bed with the heating system, where the period of time over which a certain level of direct solar radiation is available will determine the temperature at the inlet to the packed bed. 

After the experiments had been completed, the results were subjected to uncertainty analysis. The uncertainty of determining the thermo-hydraulic efficiency can be tested with the analysis of the measuring accuracy of the equipment used. The analysis of uncertainty for the equipment used in calculation of such parameters as temperature, volume flux and pressure drop was done according to the procedures described in [31]. For the calculated extended uncertainties, a coverage factor of 2 was adopted. Uncertainties, specified by the manufacturers, are: 0.1 °C for temperature, 1% for airflow in m^3^/s and 0.5 Pa for pressure drop measurement. Values of uncertainty bars were added to the characteristics represented in the next Chapter.

## 5. Model Validation

The results obtained from the balance equations described in Section 3 were compared with the results of experiments. The model included the boundary conditions obtained in experiments and the outlet temperature served as the comparative parameter. The results of analytical calculations were generated for all eight of the Nusselt number formulas. To validate the results, Equation (42) was used, which describes the deviation of outlet air temperature values obtained from the model in relation to the values obtained in the experiments:

(42) $$\delta T_{out}=\left|\frac{T_{out}-T_{out.m}}{T_{out}}\right|\cdot 100,\;\%.$$

**Figure 6:**
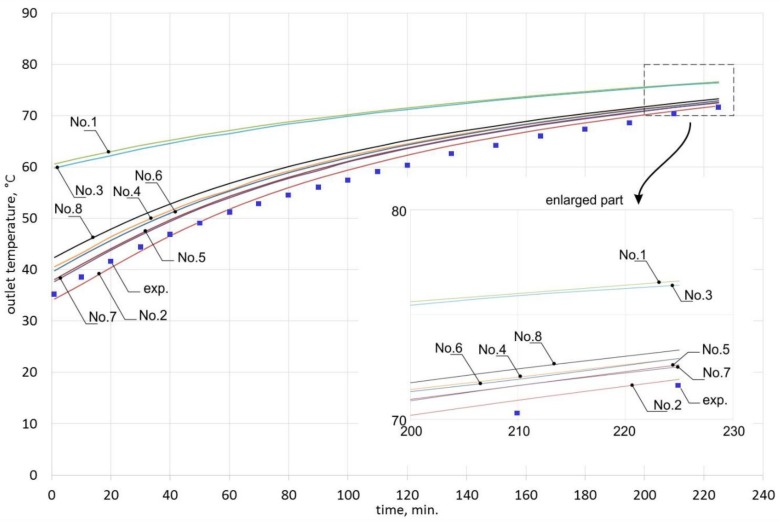
Outlet temperature for the airflow of 0.0050 m^3^/s, for each of the dimensionless equations from Table 2.

**Figure 7:**
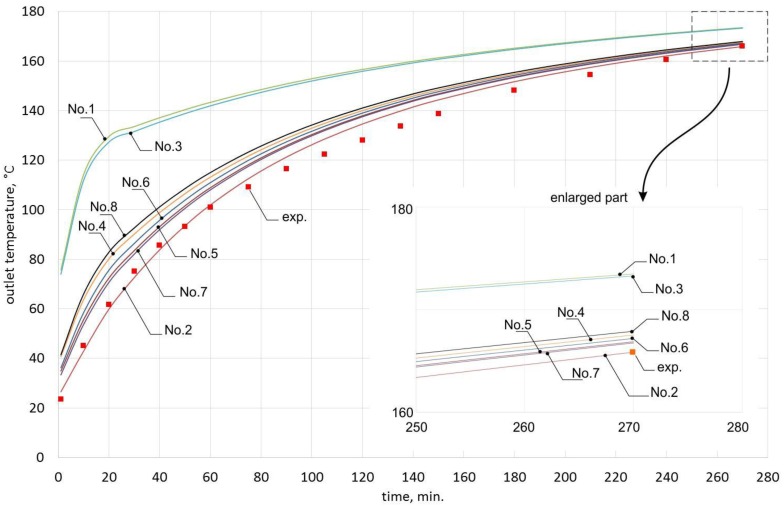
Outlet temperature for the airflow of 0.0068 m^3^/s, for each of the dimensionless equations from Table 2.

Table 3 and Table 4 show the results for the airflow of 0.0050 m^3^/s. Table 5 and Table 6 shows the results for the airflow of 0.0068 m^3^/s. The results are presented graphically in Figure 6 and Figure 7, respectively.

When analyzing the obtained results, one can observe that for the airflow of 0.0050 m^3^/s, the deviations of outlet temperature are significantly lower than for the airflow of 0.0068 m^3^/s. In the case of formula No. 1 (see Table 2), the deviations are up to 72.2% for the first airflow and up to 223.7% for the second airflow.

Figure 6 and Figure 7 show that outlet temperatures for 6 dimensionless equations of the *Nu* number are similar to experiment results. Outlet temperatures obtained from models based on formulas Nos. 1 and 3 show the changes of outlet temperature to have a completely different character.

The results obtained for formula No. 2 were closest to the experimentally obtained results. Applying this formula for the airflow of 0.0050 m^3^/s resulted in the average deviation of 2.2%, the maximum deviation of 3.6%, and the minimum deviation of 0.4%. In the case of the airflow of 0.0068 m^3^/s, these deviations were much greater: average of 3.5%, maximum of 13.2%, and minimum of 0%. Since formula No. 2 was assumed to sufficiently represent the character of changes in the analyzed process, it was selected for further analysis.

### Analysis of the Thermo-Hydraulic Efficiency for the Heat Storage Process in Ceramic Bricks

**Figure 8:**
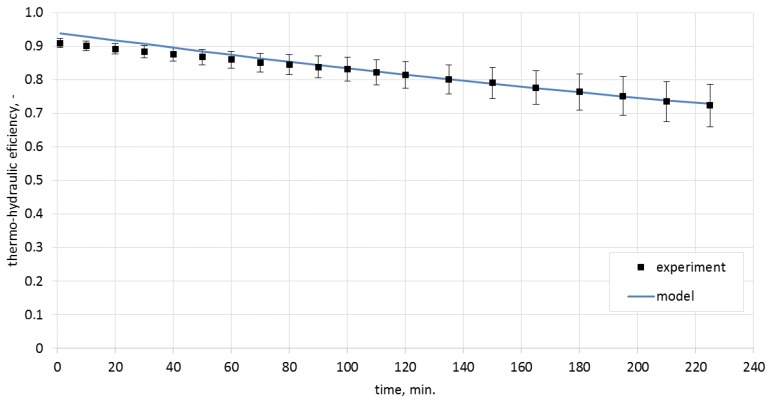
Thermo-hydraulic efficiency for the airflow rate of 0.0050 m^3^/s.

**Figure 9:**
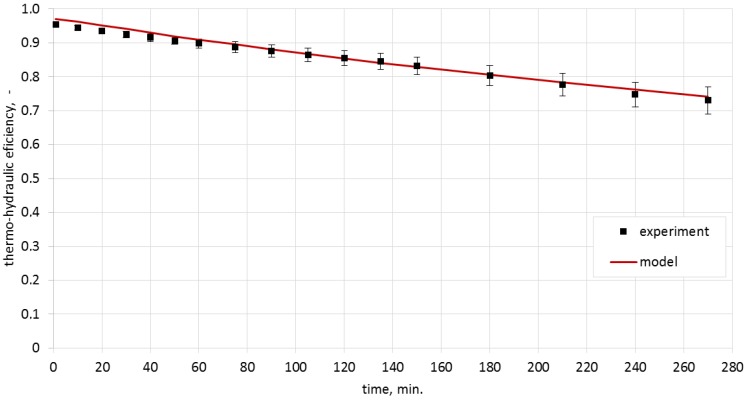
Thermo-hydraulic efficiency for the airflow rate of 0.0068 m^3^/s.

Calculating the thermo-hydraulic efficiency for the charging process required the measurement of pressure drops in the system. These were 1.7 Pa for the airflow of 0.0050 m^3^/s, and 3.0 Pa for the airflow of 0.0068 m^3^/s. The analytically determined pressure drops were, respectively, 1.8 Pa and 3.3 Pa. Efficiencies were calculated from Equation (4). The model results, along with the experimentally obtained data and with uncertainty bars, are presented in Figure 8 and Figure 9.

The analysis of the obtained characteristics allowed concluding that the process of heat storage in ceramic brick has high efficiency which, during the experiment, was in the range of 72%–93% for the airflow rate of 0.0050 m^3^/s and 74%–96% for the airflow rate of 0.0068 m^3^/s.

**Figure 10:**
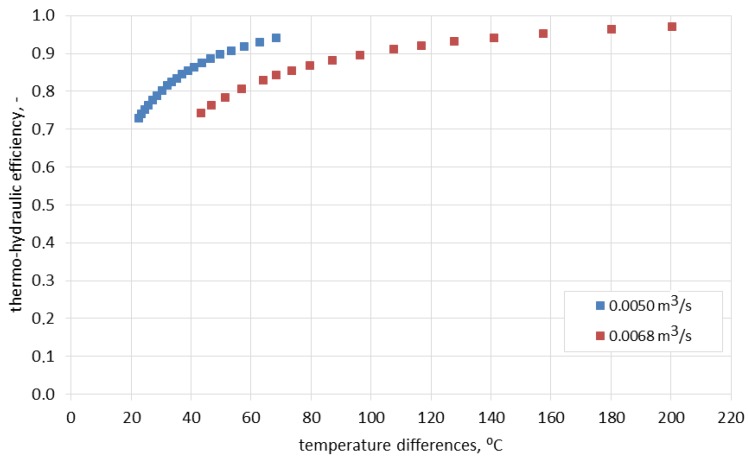
Thermo-hydraulic efficiency as a function of the difference between the inlet temperature and ceramic brick temperature.

As shown in Figure 10, one of the reasons for the change of efficiency in time is the change of the difference between the inlet temperature and the ceramic brick temperature. Notably, the thermo-hydraulic efficiency was over 70% already for the lowest temperature differences recorded during the experiment. As can be observed in Figure 9, process efficiency for lower temperature differences can be still maintained at a constant level by limiting the airflow rate. This fact is of great importance if the storage device is integrated with a heat source of variable intensity.

**Figure 11:**
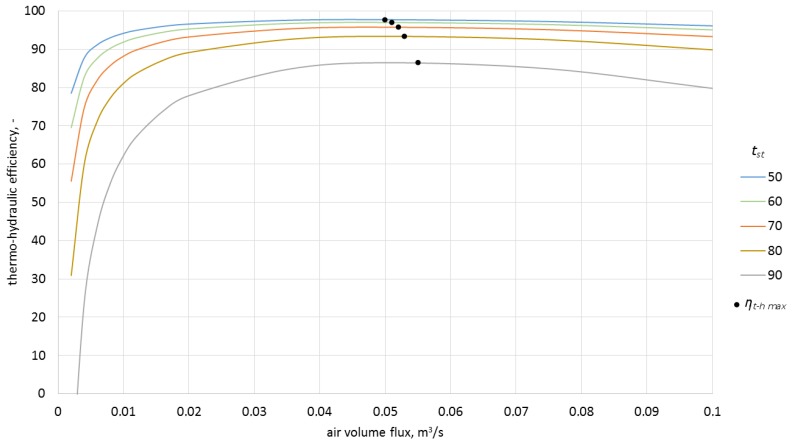
Thermo-hydraulic efficiency for the inlet temperature of 100 °C and various ceramic brick temperatures.

**Figure 12:**
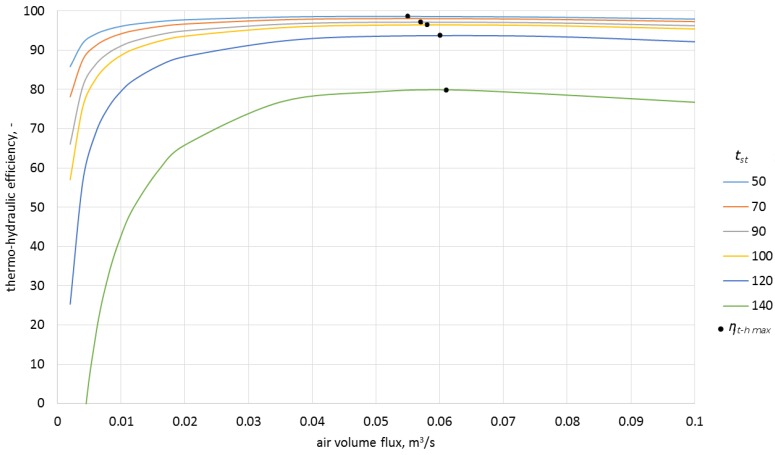
Thermo-hydraulic efficiency for the inlet temperature of 150 °C and various ceramic brick temperatures.

**Figure 13:**
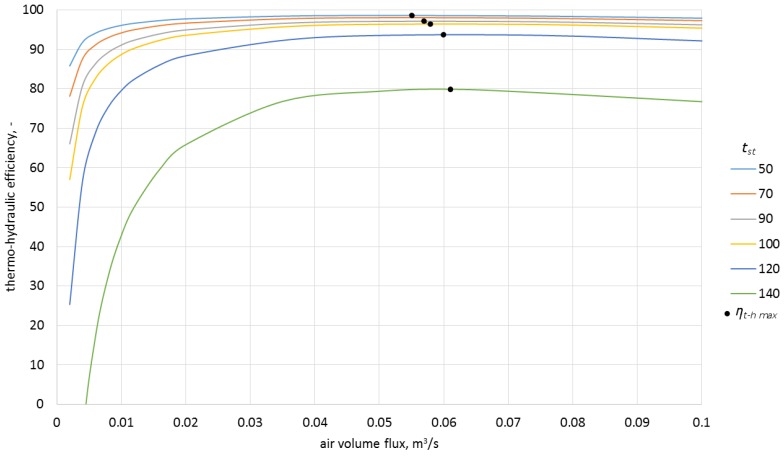
Thermo-hydraulic efficiency for the inlet temperature of 200 °C and various ceramic brick temperatures.

**Figure 14:**
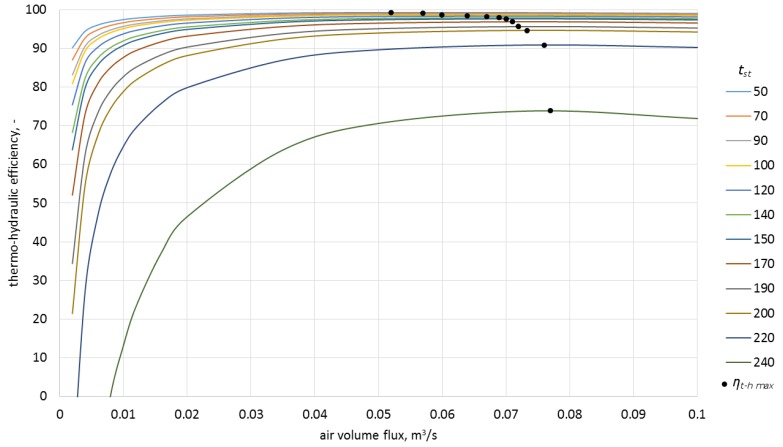
Thermo-hydraulic efficiency for the inlet temperature of 250 °C and various ceramic brick temperatures.

In order to estimate the influence of airflow rate on the efficiency of the process, the model was used to calculate the characteristics for four inlet temperatures as a function of airflow rate and various ceramic brick temperatures (Figure 11, Figure 12, Figure 13 and Figure 14).

As can be observed in the above graphs, the thermo-hydraulic efficiency in the analyzed packed bed exceeds 90% for a wide range of airflow rates. The appropriate difference between the inlet temperature and ceramic brick temperature is of importance. For the inlet temperature of up to 150 °C, this difference needed to exceed 20 °C, while for the temperatures between 150 °C and 250 °C, the difference needed to exceed 30 °C. In the analyzed case, the thermo-hydraulic efficiency reaches maximal value for airflow rate of approximately 0.05 m^3^/s. The *η_t − h max_* occurs when the airflow rate increases and the difference between the inlet temperature and the ceramic brick temperature decreases. The decrease in the thermo-hydraulic efficiency for greater airflow rates is caused by increasing pressure loss.

## 6. Conclusions

The thermo-hydraulic efficiency reaches maximal value for certain airflow rates and temperature difference. Such a maximum does not occur for the thermal efficiency, which increases together with increasing airflow rate. This is caused by the rapidly increasing pressure drop accompanied by the increasing speed of the working medium. The above fact means that the thermo-hydraulic efficiency is a good indicator of the economic effectiveness of the process.

As demonstrated in the above analyses, a system in which heat is stored in a sensible heat storage material, such as ceramic brick, should be provided with a means to control airflow rate in order to maximize the effectiveness of heat storage process, if the heat comes from a source of variable intensity, such as a concentrated solar air collector.

The developed model of the process of heat storage in a sensible heat storage material such as ceramic brick allows a precise description of heat storage phenomenon. The research results presented in this article are intended to aid the verification of a concept of using a sensible heat storage device coupled with a solar air heater, which may ensure thermal self-sufficiency to a residential house on a yearly basis. Therefore, the analyses in the next stage of research will cover the cooperation of the previously examined experimental set-ups with the solar collector and the packed bed to verify the assumed concept.

## Figures and Tables

**Table 1:** Input parameters used in the mathematical model.

**Rock bed**	Internal height	0.5 m
	Internal thickness	0.3 m
	Internal length	0.3 m
**Storage material**	Equivalent sphere diameter	0.149 m
	Specific heat	880 J/kgK
	Density	1800 kg/m^3^
	Porosity	0.4
	Temperature in bed 1	18 °C
	Temperature in bed 2	28 °C
	Mass	40.16 kg
**Air**	Volumetric flow rate 1	0.0068 m^3^/s
	Volumetric flow rate 2	0.0050 m^3^/s
	Temperature inlet 1	220 °C
	Temperature inlet 2	100 °C
	Specific heat	f(T)
	Density	f(T)
	Kinematic viscosity	f(T)
	Ambient temperature	18 °C
**Insulation**	Top insulation thickness	0.20 m
	Bottom insulation thickness	0.15 m
	Side insulation thickness	0.15 m
	Specific heat	0.039 W/mK

**Table 2:** Selected Nusselt number formulas.

No.	Dimensionless Equation		Notes	Reference
**1**	$Nu_{w}=2+0.6\cdot Re^{0.5}\cdot Pr^{1/3}$	(34)	1 ≤ Re ≤ 70,000 0.6 ≤ Pr ≤ 400	[19]
**2**	$Nu_{w}=0.8\cdot Re^{0.7}\cdot Pr^{0.33}$	(35)	500 ≤ Re ≤ 50,000	[19]
**3**	$Nu_{w}=2+0.03\cdot Re^{0.54}\cdot Pr^{\frac{1}{33}}+0.35\cdot Re^{0.58}\cdot Pr^{0.356}$	(36)	-	[26]
**4**	$Nu_{w}=2+1.8\cdot Re^{0.5}\cdot Pr^{1/3}$	(37)	100 ≤ Re Pr for typical gases and liquids	[26]
**5**	$Nu_{w}=2+1.1\cdot Re^{0.6}\cdot Pr^{1/3}$	(38)	15 ≤ Re ≤ 8500	[27]
**6**	$Nu_{w}=0.29\cdot Re^{0.8}\cdot Pr^{1/2}$	(39)	Re ≤ 2400	[28]
**7**	$Nu_{w}=2+1.354\cdot Re^{\frac{1}{2}}\cdot Pr^{\frac{1}{3}}+0.0326\cdot Re\cdot Pr^{1/2}$	(40)	60 ≤ Re	[29]
**8**	$Nu_{w}=0.437\cdot Re^{0.75}\cdot \psi^{3.35}\cdot \varepsilon^{-1.62}\cdot \left[exp\left\{29.03{\left(log\psi \right)}^{2}\right\}\right]$	(41)	$\varepsilon ={\frac{V_{b}-V_{s}}{V_{b}}}^{*}$ $\psi ={\frac{a_{s}}{a_{c}}}^{**}$	[30]

* *ε* is the filling factor of the packed bed with air, *V_b_* is the bed volume and *V_s_* is the volume of the filler material; ** ψ is the area ratio, as is the area of the sphere and ac is the area of the brick.

**Table 3:** Experiment results for the airflow of 0.0050 m^3^/s.

Experiment			Models							
			No. 1	No. 2	No. 3	No. 4	No. 5	No. 6	No. 7	No. 8
No.	*t*	*T_out_*	*T_out_*	*T_out_*	*T_out_*	*T_out_*	*T_out_*	*T_out_*	*T_out_*	*T_out_*
	min	°C	°C	°C	°C	°C	°C	°C	°C	°C
**1**	1	35.2	60.6	34.3	59.8	40.6	38.1	39.8	37.7	42.4
**2**	10	38.5	61.8	37.1	61.0	43.2	41.0	42.7	40.6	45.1
**3**	20	41.6	63.0	40.4	62.2	46.4	44.1	45.7	43.8	47.9
**4**	30	44.4	64.2	43.6	63.5	49.1	47.0	48.5	46.7	50.5
**5**	40	46.8	65.2	46.6	64.6	51.5	49.7	51.0	49.4	52.8
**6**	50	49.1	66.2	49.3	65.7	53.7	52.1	53.3	51.9	54.9
**7**	60	51.1	67.1	51.8	66.5	55.5	54.3	55.3	54.0	56.8
**8**	70	52.9	68.0	54.0	67.5	57.6	56.2	57.2	56.0	58.5
**9**	80	54.5	68.8	56.0	68.4	59.2	58.0	58.9	57.8	60.1
**10**	90	56.0	69.5	57.8	69.1	60.7	59.6	60.4	59.3	61.5
**11**	100	57.4	70.2	59.4	69.9	62.1	61.1	61.8	61.0	62.8
**12**	110	59.1	70.9	60.9	70.6	63.4	62.5	63.1	62.3	64.0
**13**	120	60.3	71.5	62.3	71.2	64.5	63.7	64.3	63.6	65.2
**14**	135	62.5	72.4	64.2	72.1	66.1	65.4	65.9	65.3	66.7
**15**	150	64.2	73.2	65.8	73.0	67.5	66.9	67.4	66.8	68.0
**16**	165	66.0	74.0	67.3	73.8	68.8	68.3	68.7	68.2	69.3
**17**	180	67.3	74.7	68.6	74.5	70.0	69.5	69.9	69.4	70.4
**18**	195	68.6	75.4	69.8	75.2	71.1	70.6	71.0	70.5	71.4
**19**	210	70.3	76.0	70.9	75.9	72.0	71.6	71.9	71.6	72.4
**20**	225	71.6	76.6	71.9	76.4	72.9	72.6	72.9	72.5	73.3

**Table 4:** Deviations for the airflow of 0.0050 m^3^/s.

Experiment			Models							
			No. 1	No. 2	No. 3	No. 4	No. 5	No. 6	No. 7	No. 8
No.	*t*	*T_out_*	*δT_out_*	*δT_out_*	*δT_out_*	*δT_out_*	*δT_out_*	*δT_out_*	*δT_out_*	*δT_out_*
	min	°C	%	%	%	%	%	%	%	%
**1**	1	35.2	72.2	2.6	69.9	15.3	8.2	13.1	7.1	20.5
**2**	10	38.5	60.5	3.6	58.4	12.2	6.5	10.9	5.5	17.1
**3**	20	41.6	51.4	2.9	49.5	11.5	6.0	9.9	5.3	15.1
**4**	30	44.4	44.6	1.8	43.0	10.6	5.9	9.2	5.2	13.7
**5**	40	46.8	39.3	0.4	38.0	10.0	6.2	9.0	5.6	12.8
**6**	50	49.1	34.8	0.4	33.8	9.4	6.1	8.6	5.7	11.8
**7**	60	51.1	31.3	1.4	30.1	8.6	6.3	8.2	5.7	11.2
**8**	70	52.9	28.5	2.1	27.6	8.9	6.2	8.1	5.9	10.6
**9**	80	54.5	26.2	2.8	25.5	8.6	6.4	8.1	6.1	10.3
**10**	90	56.0	24.1	3.2	23.4	8.4	6.4	7.9	5.9	9.8
**11**	100	57.4	22.3	3.5	21.8	8.2	6.4	7.7	6.3	9.4
**12**	110	59.1	20.0	3.0	19.5	7.3	5.8	6.8	5.4	8.3
**13**	120	60.3	18.6	3.3	18.1	7.0	5.6	6.6	5.5	8.1
**14**	135	62.5	15.8	2.7	15.4	5.8	4.6	5.4	4.5	6.7
**15**	150	64.2	14.0	2.5	13.7	5.1	4.2	5.0	4.0	5.9
**16**	165	66.0	12.1	2.0	11.8	4.2	3.5	4.1	3.3	5.0
**17**	180	67.3	11.0	1.9	10.7	4.0	3.3	3.9	3.1	4.6
**18**	195	68.6	9.9	1.7	9.6	3.6	2.9	3.5	2.8	4.1
**19**	210	70.3	8.1	0.9	8.0	2.4	1.8	2.3	1.8	3.0
**20**	225	71.6	7.0	0.4	6.7	1.8	1.4	1.8	1.3	2.4
deviation %		avg.	27.6	2.2	26.7	7.7	5.2	7.0	4.8	9.5
		max.	72.2	3.6	69.9	15.3	8.2	13.1	7.1	20.5
		min.	7.0	0.4	6.7	1.8	1.4	1.8	1.3	2.4

**Table 5:** Experiment for the airflow of 0.0068 m^3^/s.

Experiment			Models							
			No. 1	No. 2	No. 3	No. 4	No. 5	No. 6	No. 7	No. 8
No.	*t*	*T_out_*	*T_out_*	*T_out_*	*T_out_*	*T_out_*	*T_out_*	*T_out_*	*T_out_*	*T_out_*
	min	°C	°C	°C	°C	°C	°C	°C	°C	°C
**1**	1	23.4	75.7	26.5	74.0	40.7	34.9	36.2	33.4	41.5
**2**	10	45.2	114.3	43.0	111.9	63.8	55.7	58.6	53.8	66.1
**3**	20	61.7	129.6	59.9	127.3	80.2	72.5	75.7	70.8	82.9
**4**	30	75.2	133.6	72.7	131.6	90.2	83.6	86.5	82.1	92.7
**5**	40	85.7	137.1	83.9	135.4	98.9	93.2	95.8	92.0	101.2
**6**	50	93.2	140.4	93.5	138.8	106.5	101.6	103.9	100.6	108.6
**7**	60	101.0	143.3	101.9	141.9	113.1	108.9	110.9	108.0	115.0
**8**	75	109.1	147.3	112.5	146.1	121.7	118.2	119.9	117.5	123.3
**9**	90	116.4	150.8	121.2	149.8	128.8	125.9	127.4	125.3	130.2
**10**	105	122.4	153.9	128.5	153.0	134.8	132.4	133.7	131.9	136.0
**11**	120	128.1	156.6	134.6	155.9	140.0	137.9	139.0	137.5	141.1
**12**	135	133.6	159.1	139.8	158.4	144.5	142.7	143.6	142.3	145.4
**13**	150	138.7	161.3	144.3	160.8	148.4	146.8	147.6	146.5	149.2
**14**	180	148.1	165.2	151.7	164.8	154.8	153.6	154.2	153.4	155.4
**15**	210	154.4	168.4	157.4	168.1	159.9	159.0	159.5	158.8	160.5
**16**	240	160.5	171.1	162.1	170.9	164.1	163.3	163.7	163.2	164.5
**17**	270	165.9	173.5	165.9	173.3	167.5	166.9	167.2	166.8	167.9

**Table 6:** Deviations for the airflow of 0.0068 m^3^/s.

Experiment			Models							
			No. 1	No. 2	No. 3	No. 4	No. 5	No. 6	No. 7	No. 8
No.	*t*	*T_out_*	*δT_out_*	*δT_out_*	*δT_out_*	*δT_out_*	*δT_out_*	*δT_out_*	*δT_out_*	*δT_out_*
	min	°C	%	%	%	%	%	%	%	%
**1**	1	23.4	223.7	13.2	216.2	74.1	49.2	54.9	42.6	77.3
**2**	10	45.2	152.8	4.9	147.6	41.1	23.3	29.6	19.0	46.2
**3**	20	61.7	110.0	2.8	106.4	30.0	17.5	22.6	14.7	34.4
**4**	30	75.2	77.6	3.3	74.9	19.9	11.1	15.0	9.2	23.3
**5**	40	85.7	60.0	2.1	58.0	15.4	8.8	11.8	7.3	18.1
**6**	50	93.2	50.6	0.4	48.9	14.3	9.0	11.5	7.9	16.5
**7**	60	101.0	41.9	0.9	40.5	12.0	7.8	9.8	7.0	13.9
**8**	75	109.1	35.0	3.2	33.9	11.5	8.3	9.9	7.7	13.0
**9**	90	116.4	29.5	4.2	28.7	10.7	8.2	9.4	7.7	11.8
**10**	105	122.4	25.7	5.0	25.0	10.2	8.2	9.2	7.8	11.1
**11**	120	128.1	22.3	5.1	21.7	9.3	7.7	8.5	7.4	10.1
**12**	135	133.6	19.1	4.7	18.6	8.1	6.8	7.5	6.5	8.8
**13**	150	138.7	16.3	4.1	15.9	7.0	5.8	6.4	5.6	7.5
**14**	180	148.1	11.5	2.4	11.2	4.5	3.7	4.1	3.6	5.0
**15**	210	154.4	9.1	2.0	8.9	3.6	2.9	3.3	2.8	3.9
**16**	240	160.5	6.6	1.0	6.5	2.2	1.7	2.0	1.7	2.5
**17**	270	165.9	4.6	0.0	4.5	1.0	0.6	0.8	0.5	1.2
deviation %		avg.	52.7	3.5	51.0	16.2	10.6	12.7	9.3	17.9
		max.	223.7	13.2	216.2	74.1	49.2	54.9	42.6	77.3
		min.	4.6	0.0	4.5	1.0	0.6	0.8	0.5	1.2
